# The analgesic efficacy of ultrasound-guided transversus abdominis plane block for retroperitoneoscopic renal surgery: a randomized controlled study

**DOI:** 10.1186/s12871-019-0850-3

**Published:** 2019-10-18

**Authors:** Xue Li, Zhen-Zhen Xu, Xue-Ying Li, Ting-Ting Jiang, Zeng-Mao Lin, Dong-Xin Wang

**Affiliations:** 1 Department of Anesthesiology, Peking University First Hospital, No. 7 Xishiku Street, Xicheng District, Beijing, 100034 China; 2 Department of Biostatics, Peking University First Hospital, No. 7 Xishiku Street, Xicheng District, Beijing, 100034 China

**Keywords:** Transversus abdominis plane block, Analgesia, Retroperitoneoscopic renal surgery, Postsurgical recovery

## Abstract

**Background:**

Ultrasound-guided lateral transversus abdominis plane (TAP) block can provide definite analgesia to the anterior abdominal wall. However, whether this method is useful in renal surgery through the lateral abdominal wall pathway remains unknown. The study aimed to evaluate the analgesic efficacy of lateral TAP block for retroperitoneoscopic partial or radical nephrectomy.

**Method:**

In this prospective, randomized, double-blind, placebo-controlled trial, eligible patients were randomized into two groups. After anaesthesia induction, ultrasound-guided lateral TAP block was performed with either 30 ml of 0.4% ropivacaine (Group T) or an equivalent volume of normal saline (Group C). The primary outcomes were opioid consumption during surgery and in the first 24 h after surgery. Secondary outcomes included postsurgical pain intensity immediately awakening from anaesthesia and at 0.5, 1, 2, 6, 12, and 24 h after surgery, as well as recovery variables including the incidence of postoperative nausea and vomiting (PONV), sleep quality, time to first ambulation, drainage and length of hospital stay.

**Results:**

A total of 104 patients were enrolled and randomized (53 in Group T and 51 in Group C). Laparoscopic surgery was converted to open surgery in one patient of Group T; this patient was excluded from the outcome analysis. The opioid consumption during surgery (intravenous morphine equivalent dose: median 35.0 mg [interquartile range 18.0, 49.6] in Group C vs. 40.3 mg [20.9, 59.0] in Group T, *P* = 0.281) and in the first 24 h after surgery (10.8 mg [7.8, 21.7] in Group C vs. 13.2 mg [8.0, 26.6] in Group T, *P* = 0.311) did not differ significantly between groups. There were no significant differences between groups regarding the pain intensity at all time points after surgery and the recovery variables (all *P* > 0.05).

**Conclusions:**

Our results showed that, in patients undergoing retroperitoneoscopic renal surgery, preoperative lateral TAP did not decrease intra- and postoperative opioid consumption, nor did it relieve pain intensity or promote postoperative recovery in the first 24 h after surgery. However, the trial might be underpowered.

**Trial registration:**

This study was registered on November 4, 2017, in the Chinese Clinical Trail Registry with the identification number ChiCTR-INR-17013244.

## Background

For laparoscopic renal surgery, the retroperitoneal approach is an alternative pathway of the transperitoneal approach. The overall outcomes of both approaches, such as the rates of perioperative complications, positive surgical margin and postoperative recurrence, are similar; whereas the retroperitoneoscopic approach is advantageous in terms of easier hilar control and shorter total operative time, especially in patients with a past history of intraperitoneal procedures or with a posteriorly located renal tumour [1–3]. Therefore, the retroperitoneoscopic approach is the most popular approach for radical or partial nephrectomy in Peking University First Hospital. Multimodal analgesia including nerve block is advocated to improve early recovery after renal surgery [4, 5].

Transversus abdominis plane (TAP) block is an effective regional anaesthetic technique that blocks neural afferents of the T6-L1 spinal nerves innervating the anterolateral abdominal wall [6]. Since the original report by Rafi [7], there have been a plethora of studies on this block and variations of the original approach, among which lateral TAP is the most commonly used approach in abdominal surgery, with its dermatomal sensory block covering T10 to L1 [8]. A recent systematic review demonstrated that TAP block had definite analgesic efficiency for some kinds of lower abdominal surgeries, such as gynaecological surgery, caesarean section and hernia repair, but not urologic surgery [9]. However, it was worth noting that there was a high heterogeneity among the urologic studies included in that review. In addition, some studies focusing on renal surgery were missed.

To our knowledge, only three randomized controlled trials compared the effect of lateral TAP block with placebo in laparoscopic live-donor nephrectomy [10–12]. All of these found that TAP block surely reduced postoperative pain severity and opioid requirements. However, this conclusion can’t be extrapolated to retroperitoneal laparoscopic renal surgery (RLRS) of which the main incision is completely different from those of live-donor nephrectomy. Theoretically, the dermatomes of lateral TAP block can only partially cover the incision in RLRS. Therefore, whether it could reduce opioid consumption and subjective pain intensity, and ultimately promote postoperative recovery in patients undergoing RLRS remains unknown. The purpose of this study was to determine whether lateral TAP block could provide effective analgesia and improve recovery in patients undergoing RLRS.

## Methods

### Study design

This prospective, randomized, double-blinded trial was approved by the Biomedical Research Ethics Committee of Peking University First Hospital (2017–1398). It was registered at http://www.chictr.org.cn with an identification number of ChiCTR-INR-17013244. Written informed consent was obtained from each patient. The study adhered to the CONSORT guidelines.

### Participants

Potential participants were screened the day before surgery. Patients aged between 18 and 70 years and scheduled to undergo elective laparoscopic radical or partial nephrectomy through the retroperitoneal approach were included. Patients who met any of the following criteria were excluded: (1) chronic opioid addiction and/or use of other kinds of analgesic drugs for more than 3 months; (2) inability to communicate due to severe dementia, language barrier, or end-stage disease; (3) allergic to local anaesthetics; (4) nerve block contraindication such as an infection in the puncture site or severe coagulation dysfunction; and (5) refusal to participate in the study. Patients who were enrolled for this trial were taught how to evaluate pain intensity by using the numeric rating scale (NRS, an 11-point scale where 0 indicates no pain and 10 indicates the worst pain) and how to use a patient-controlled analgesia (PCA) device.

### Anaesthesia management and surgical technique

All patients were managed according to a standardized anaesthetic protocol. Anaesthesia was induced with sufentanil, propofol and etomidate. Endotracheal intubation was facilitated with cisatracurium or rocuronium. Anaesthesia was maintained with continuous infusion of propofol, remifentanil (and intermittent sufentanil) or sufentanil, with or without dexmedetomidine; the aim was to maintain the BIS values between 40 and 60, and the mean arterial pressure and heart rate within 20% of the preoperative values. At 30 min before the end of surgery, 50 mg of flurbiprofen axetil and 5 mg of tropisetron were administered intravenously. After emergence from anaesthesia, all patients were monitored in the post-anaesthesia care unit (PACU) for at least 1 h before transferred to the general ward.

**Fig. 1:**
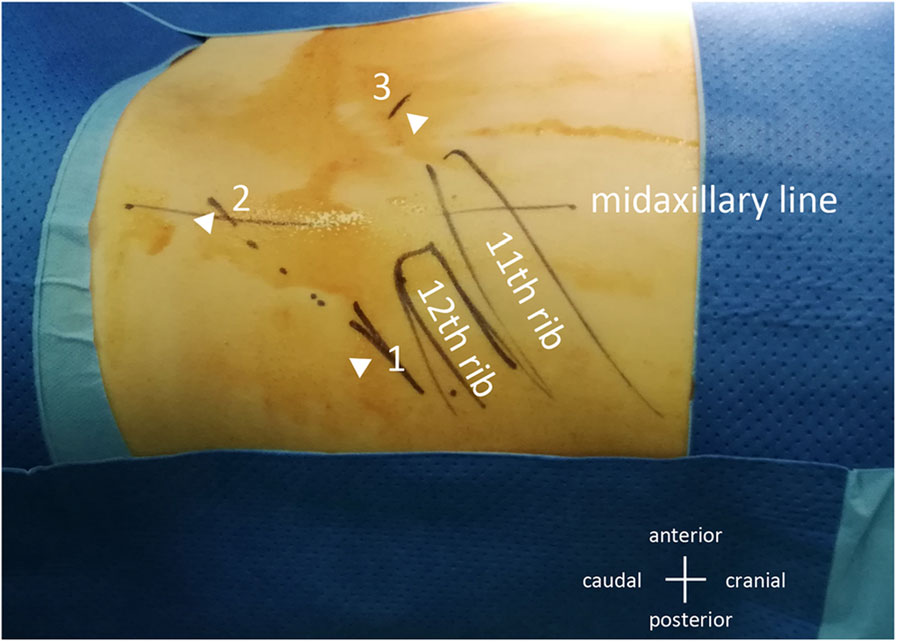
The three trocar sites for retroperitoneoscopic renal surgery

The retroperitoneal laparoscopic procedure was usually performed through three ports. The primary port was invariably placed through the incision made for the creation of the working space, which was just below the tip of the 12th rib on the posterior axillary line. The secondary port was placed 2 cm above the iliac crest on the midaxillary line. The third port was placed under the costal margin on the anterior axillary line. In case of radical nephrectomy, the initial incision was extended ventrally for kidney removal. The pneumoperitoneum was maintained at approximately 12–14 mmHg throughout the procedure (Fig. 1).

For all patients, a standard postoperative pain management was provided, i.e., a PCA pump, which was established with 1.25 μg/ml sufentanil and programmed to administer a background rate of 0.5 ml/h and an on-demand bolus of 4 ml every 10 min, together with a rigorous rescue analgesia plan. The target was to maintain the NRS pain score below 4. In the PACU, regular pain evaluation was performed every 30 min. If the NRS score was higher than 4, a PCA bolus of 4 ml was administered first, and pain was evaluated 5 min later. If the NRS score remained higher than 4, another 3–5 μg sufentanil was administered intravenously according to the patient’s body weight. No more rescue analgesics were administered if the NRS score decreased to 4 or less. In the general ward, pain evaluation was performed at 2, 6, 12, and 24 h after surgery; in addition, patients were instructed to request additional analgesia in case of breakthrough pain. Pain control measures were similar to those in the PACU, except that morphine (3–5 mg) was administered instead of sufentanil. NSAIDS or other analgesics could also be administered according to the surgeons’ prescription.

### Randomization and intervention

Stratified randomization with a block size of 4 was performed using the SAS statistical package version 9.3 (SAS Institute, Cary, NC, USA) by a biostatistician (XLN) who was not involved in the data management and statistical analyses. Stratification was performed according to the planned type of surgery, i.e., radical or partial nephrectomy. The randomization results were then sealed in sequentially numbered envelopes, transferred to a study coordinator (TTJ) with the Good Clinical Practice (GCP) certification and stored at the site of the investigation until the end of the study.

The day before surgery, an investigator (ZML) screened potential participants and recruited patients after obtaining written informed consents. On the day of surgery, the study coordinator opened the envelopes consecutively according to the recruitment sequence and prepared the study drugs for each patient, but did not participate in the rest of the trial. All study drugs were provided as clear aqueous solutions in the same 20 ml syringes for TAP block. In this way patients were randomly assigned into two groups: patients in Group T received 30 ml of 0.4% ropivacaine, while those in Group C received an equivalent amount of normal saline. Apart from the study drugs used for TAP block, other perioperative management was identical in both groups. All health-care team members, investigators, and patients themselves were fully blinded to the group assignments throughout the study period.

**Fig. 2:**
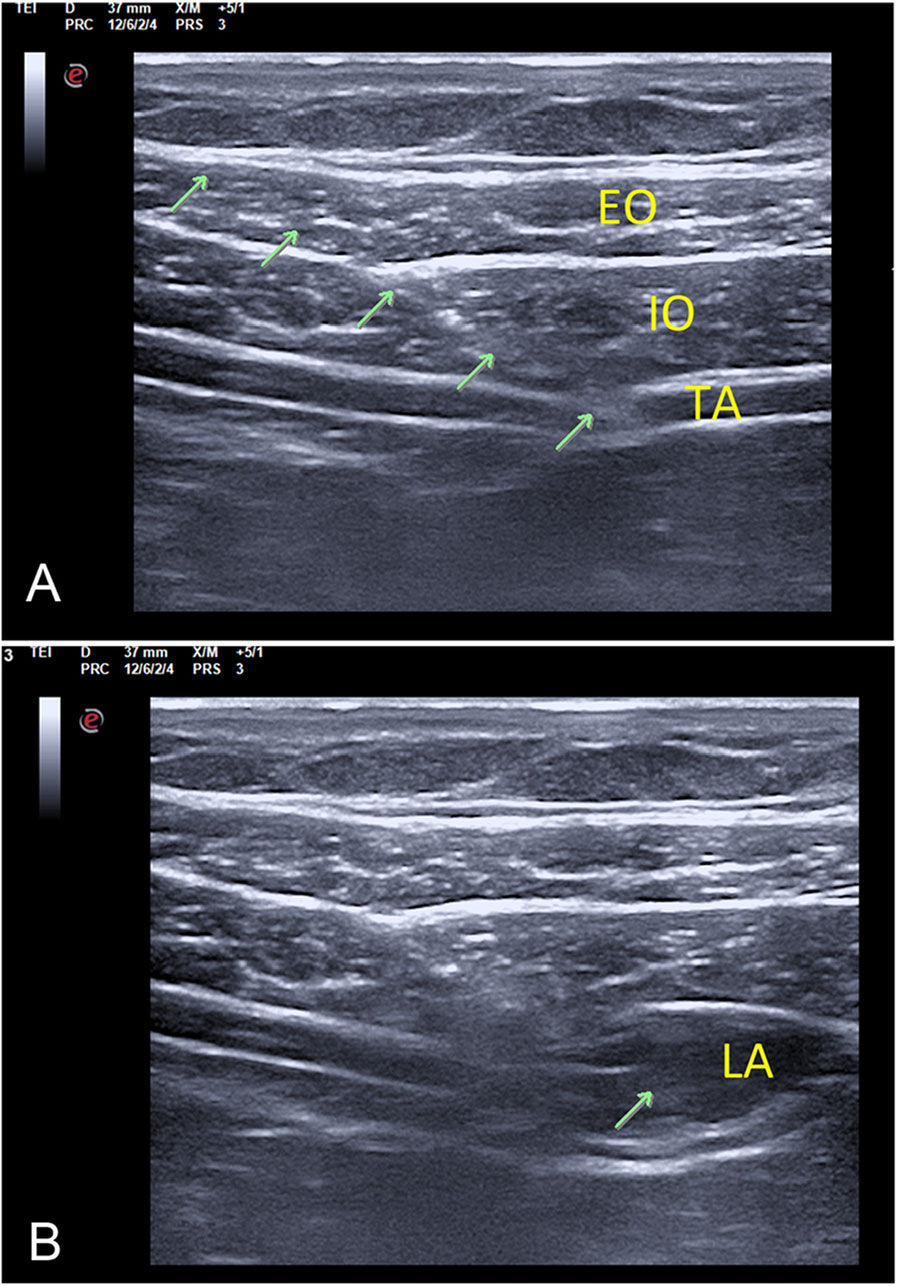
Sonography of the lateral TAP block, indicating the ultrasound anatomical structure (**a**) and the spread of local anaesthetic (**b**). The green arrow indicates the needle trajectory; EO: external oblique; IO: internal oblique; TA: transversus abdominis; LA: local anaesthetic

Ultrasound-guided TAP block was performed by two experienced anaesthetists (DH and HK) immediately after the induction of anaesthesia and approximately 15 min before skin incision. With the patient in the supine position, the ultrasound probe was placed at the midaxillary line between the lower costal margin and the iliac crest. At this point, the plane between the internal oblique and transverse abdominal muscles was identified (Fig. 2a). A special needle used for nerve block (80 mm or 100 mm, Stimuplex D, Germany) was inserted using an in-plane technique in the anteroposterior direction. After aspiration, to avoid inadvertent intravascular injection and abdominal paracentesis, an injection with 2 ml of normal saline was used to ensure correct positioning of the needle. The prepared study drug was then injected into this plane. Successful study drug injection was defined as the appearance of a hypoechoic ellipsoid with well-defined margins on ultrasonic imaging (Fig. 2b).

### Follow-up schedule and outcomes

Investigators (XL, ZZX, and ZML) who were blinded to the study group assignment were in charge of the perioperative data collection. Patients were followed-up at several time points in the first 24 h after surgery. In addition, the electronic medical record was reviewed to obtain necessary data.

The primary outcomes were opioid consumption (intravenous morphine equivalent dose) during surgery and within the first 24 h after surgery. The secondary outcomes included the following: (1) the NRS pain scores both at rest and with coughing immediately awakening from anaesthesia and at 0.5, 1, 2, 6, 12 and 24 h after surgery; (2) time to the first bolus demand in the PCA system, as well as the numbers of required and administered bolus; (3) time to the first rescue analgesic, as well as its use and frequency in addition to the PCA system; (4) the incidence of postoperative nausea and vomiting (PONV) within 24 h after surgery and the use of antiemetics; (5) subjective sleep quality on the night of surgery evaluated by the NRS (an 11-point scale where 0 indicates the best sleep quality and 10 indicates the worst sleep experience); (6) time to the first ambulation after surgery; (7) drainage during the first 24 h after surgery; and (8) the length of hospital stay after surgery.

Safety outcomes were monitored from the beginning of anaesthesia until 24 h after surgery. The adverse events associated with TAP block included but not limited to the following: numbness in the lower extremities, haematoma and bleeding in the needle trajectory, visceral organ injury, anaphylaxis, local anaesthetic toxicity. Other perioperative adverse events were also documented.

### Statistical analysis

#### Sample size estimation

According to previous studies [10, 12, 13], the use of TAP block decreased opioid consumption by 13.5–45.3% compared with the placebo during the first 24 h after surgery. We conservatively assumed that opioid consumption would be reduced by 10% in the TAP block group. Sample size calculation was performed based on the previous data obtained from our clinical follow-up system, which showed that the total consumption of sufentanil (within 24 h after surgery) in patients who underwent RLRS without TAP block was 36.5 ± 5.4 μg. With the significance and power set at 0.05 (two-sided) and 90%, respectively, the sample size required to detect differences was 94 patients. Taking into account a drop-out rate of approximately 10%, we planned to enrol 104 patients. Sample size calculation was performed with the PASS 11.0 software (Stata Corp. LP, College Station, TX).

#### Outcome analyses

Normally distributed continuous variables are expressed as the mean ± standard deviation and were compared using a two-tailed Student’s t-test. Non-normally distributed continuous variables and ordinal data are expressed as medians (interquartile range) and were compared using the Mann-Whitney U test. Categorical variables are expressed as numbers (percentages) and were compared with Chi-squared analysis or Fischer’s exact test. Time-to-event data were analysed by the Kaplan-Meier estimator, with the difference between groups compared by the log-rank test. Per-protocol analysis was performed. Two-sided *P* values of less than 0.05 were regarded as statistically significant. All statistical analyses were performed with the SPSS statistical package version 25.0 (IBM SPSS Inc., Chicago, IL, USA).

## Results

**Fig. 3:**
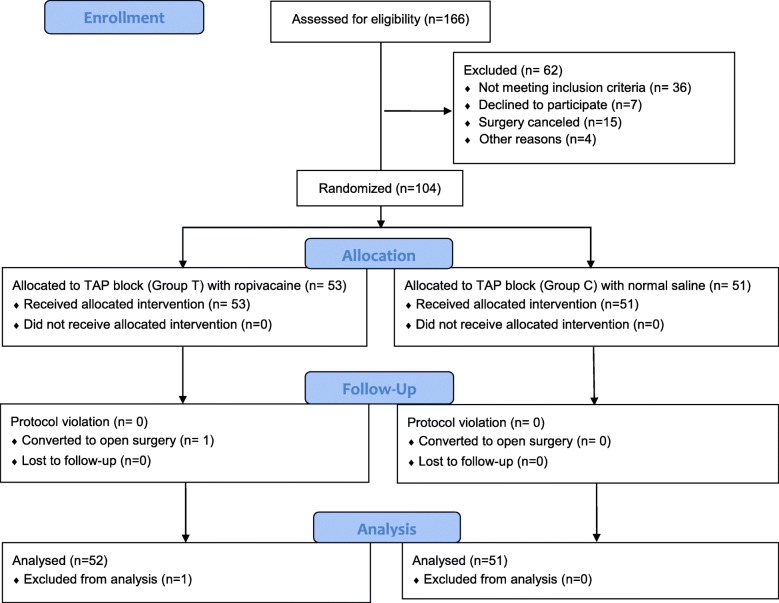
Flow diagram of the study

From January 1, 2018, to March 20, 2018, 166 patients were screened for eligibility; of these, 130 met the inclusion/exclusion criteria, 104 gave consents and were randomized into the study, with 53 patients in Group T and 51 in Group C. During surgery, one patient developed major haemorrhage and was converted to open surgery. This patient was excluded from the per-protocol analysis. Flow diagram of the study was shown in Fig. 3 and original dataset was listed as Additional file 1.

No patient received opioid treatment before surgery. The two groups were comparable regarding the demographic or baseline characteristics except that the percentage with diabetes mellitus was higher in Group T (*P* = 0.041; Table 1). Intraoperative variables, including durations of anaesthesia and surgery, use and dose of dexmedetomidine, and estimated blood loss, did not differ significantly between groups (Table 2).

**Table 1:** Demographic and baseline characteristics

	Group C (*n* = 51)	Group T (*n* = 52)	*P* value
Age, year	51.1 ± 11.1	51.9 ± 10.3	0.717
Body mass index, kg/ m^2^	24.8 ± 3.3	24.8 ± 3.8	0.929
Male	31 (60.8%)	32 (61.5%)	0.937
Type of surgery			
Radical nephrectomy	24 (47.1%)	24 (46.2%)	0.927
Partial nephrectomy	27 (52.9%)	28 (53.8%)	
ASA class			
I	27 (52.9%)	24 (46.2%)	0.421
II	24 (47.1%)	27 (51.9%)	
III	0 (0.0%)	1 (1.9%)	
NYHA class			
I	51 (100%)	50 (96.2%)	0.495
II	0 (0.0%)	2 (3.8%)	
Comorbidities			
Stoke	2 (3.9%)	4 (7.7%)	0.692
Hypertension	19 (37.3%)	16 (30.8%)	0.487
Coronary artery disease	1 (2.0%)	3 (5.8%)	0.624
Diabetes Mellitus	3 (5.9%)	10 (19.2%)	0.041
Asthma and/or COPD	2 (3.9%)	0 (0.0%)	0.243
Previous abdominal or back surgery	14 (27.5%)	10 (19.2%)	0.324

Data are presented as mean ± standard deviation or number (%)

*ASA* America Society of Anaesthesiologists, *NYHA* New York Heart Association, *COPD* chronic obstructive pulmonary disease

**Table 2:** Intraoperative data

	Group C (*n* = 51)	Group T (*n* = 52)	*P* value
Duration of anaesthesia, min	144 (122, 168)	140 (126, 165)	0.805
Duration of surgery, min	78 (59, 112)	79 (64, 102)	0.934
Use of dexmedetomidine	20 (39.2%)	13 (25.0%)	0.122
Dose of dexmedetomidine, μg	30 (23, 39) (*n* = 20)	30 (22, 50) (*n* = 13)	0.785
Estimated blood loss, ml	50 (50, 50)	50 (50, 50)	0.387

Data are presented as median (interquartile range) or number (%)

The opioid consumption during surgery (intravenous morphine equivalent dose: median 35.0 mg [interquartile range 18.0, 49.6] in Group C vs. 40.3 mg [20.9, 59.0] in Group T, *P* = 0.281) and in the first 24 h after surgery (10.8 mg [7.8, 21.7] in Group C vs. 13.2 mg [8.0, 26.6] in Group T, *P* = 0.311) did not differ significantly between groups. Stratified analysis did not find any significant differences regarding intraoperative and postoperative morphine equivalent dose between the two groups in patients receiving either the partial or radical nephrectomy (Table 3).

**Table 3:** Effectiveness outcomes

	Group C (*n* = 51)	Group T (*n* = 52)	Estimated effects (95% CI) ^a^	*P* value
Opioid consumption during surgery				
Sufentanil, μg	20 (15, 38)	23 (20, 30)	Median D = 0 (−5, 5)	0.685
Remifentanil, μg	600 (502, 794) (*n* = 33)	607 (428, 818) (*n* = 39)	Median D = 0 (− 119, 120)	0.977
Morphine equivalent dose, mg	35.0 (18.0, 49.6)	40.3 (20.9, 59.0)	Median D = 4.4 (−3.6, 13.4)	0.281
Morphine equivalent dose, mg/kg	0.58 (0.29, 0.77)	0.59 (0.30, 0.87)	Median D = 0.05 (−0.06, 0.18)	0.326
Opioid consumption within 24 h after surgery				
Sufentanil, μg	33 (23, 65)	40 (24, 80)	Median D = 4 (−4, 14)	0.311
Morphine equivalent dose, mg	10.8 (7.8, 21.7)	13.2 (8.0, 26.6)	Median D = 1.2 (−1.3, 4.8)	0.311
Morphine equivalent dose, mg/kg	0.16 (0.12, 0.31)	0.19 (0.12, 0.39)	Median D = 0.02 (−0.02, 0.07)	0.252
Laparoscopic partial nephrectomy	(*n* = 27)	(*n* = 28)		
Intraoperative MED, mg	41.2 (14.0, 49.6)	38.0 (19.0, 60.4)	Median D = 4.4 (−10.6, 16.6)	0.480
Postoperative MED within 24 h, mg	10.1 (7.4, 19.8)	17.7 (8.4, 26.1)	Median D = 3.1 (−0.3, 10)	0.070
Laparoscopic radical nephrectomy	(*n* = 24)	(n = 24)		
Intraoperative MED, mg	30.0 (19.2, 52.7)	40.7 (26.9, 53.8)	Median D = 5.5 (−6.8, 18.7)	0.370
Postoperative MED within 24 h, mg	14.5 (8.2, 25.0)	10.6 (8.0, 27.8)	Median D = -0.9 (−6.0, 4.4)	0.773
Data from the PCA system				
Number of required bolus	4 (1, 10)	6 (1, 15)	Median D = 1 (−1, 4)	0.335
Number of administered bolus	3 (1, 10)	5 (1, 12)	Median D = 1 (−1, 3)	0.338
Time to first required bolus, hour ^b^	1.7 (0.4, 3.0)	6.0 (2.8, 9.2)	HR = 1.46 (0.93, 2.30)	0.088
Data of rescue analgesia within 24 h after surgery				
Percentage of rescue analgesics	12 (23.5%)	19 (36.5%)	OR = 0.53 (0.23, 1.26)	0.153
Frequency of rescue analgesics	2 (1, 3)	1 (1, 4)	Median D = 0 (−1, 0)	0.306
Time to first rescue analgesics, hour ^b^	21.0 (14.4, 27.6)	25.0 (19.3, 30.7)	HR = 1.08 (0.52, 2.24)	0.843

Data are presented as median (interquartile range) or number of patient (%), unless otherwise indicated

*D* difference, *MED* morphine equivalent dose, *PCA* patient controlled analgesia

^a^Calculated as Group T vs. or minus Group C

^b^Data were analysed by Kaplan-Meier analysis and compared by log-rank test; the results are presented as the median (95% confidence interval)

**Fig. 4:**
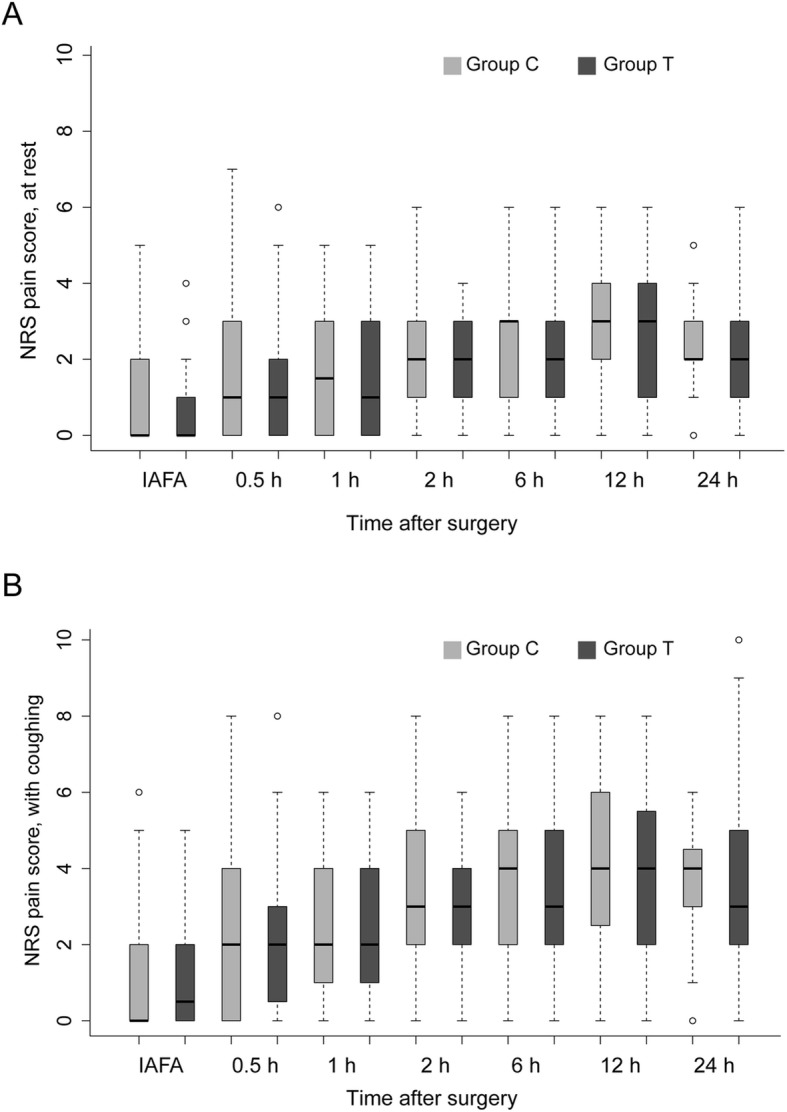
NRS pain score at rest (**a**) or with coughing (**b**) within 24 h after surgery. There were no significant differences between the two groups. IAFA: immediate awakening from anaesthesia

There were no significant differences between groups regarding the numbers of required and administered bolus, as well as the time to the first required bolus from the PCA system (*P* = 0.335, 0.338 and 0.088, respectively). There were no significant differences between groups regarding the percentage and frequency of rescue analgesics, as well as the time to first dose rescue analgesics in addition to the PCA system (*P* = 0.153, 0.306, and 0.843, respectively) (Table 3). Post-surgical pain scores at those abovementioned time points were similar between the two groups both at rest and with coughing (all *P* > 0.05) (Fig. 4a and b).

As for postoperative recovery in the first 24 h, the incidence of PONV, percentage of antiemetic therapy, subject sleep quality, time to the first ambulation, volume of drainage, and length of stay in hospital after surgery were not significantly different between the two groups (all *P* > 0.05) (Table 4).

**Table 4:** Comparisons of recovery variables

	Group C (*n* = 51)	Group T (*n* = 52)	*P* value
PONV within 24 h	15 (29.4%)	17 (32.7%)	0.719
Use of antiemetics within 24 h	10 (19.6%)	8 (15.4%)	0.573
NRS subject sleep quality, score ^a^	5 (2, 7)	4 (2, 7)	0.717
Time to first ambulation, hour ^b^	19.5 (17.8, 21.2)	20.0 (18.2, 21.8)	0.314
Volume of drainage within 24 h, ml	30 (0, 55)	35 (0, 90)	0.248
LOS in hospital after surgery, day ^b^	4.0 (3.7, 4.3)	4.0 (3.6, 4.4)	0.754

Data are presented as number (%) or median (interquartile range)

*PONV* postoperative nausea and vomiting, *NRS* numeric rating scale, *LOS* length of stay

^a^Subjective sleep quality on the night of surgery

^b^Data were analyzed by Kaplan-Meier analysis and compared by log-rank test; the results are presented as median (95% confidence interval)

No adverse events related to the TAP block technique were observed in either group. One patient in Group T developed major haemorrhage during surgery (estimated blood loss of 6500 ml), one patient in Group C developed emergence delirium. Perioperative management was uneventful in other patients.

## Discussion

To our knowledge, this was the first study investigating the efficiency of lateral TAP block in patients undergoing RLRS. Our results showed that preoperative lateral TAP block did not decrease intra- and postoperative opioid consumption, nor did it relieve pain intensity or promote postoperative recovery early after surgery. Our study added new evidence to the current knowledge of analgesic measures for laparoscopic urological surgery.

Our results conflicted with those of previous studies. For example, both Parikh et al. and Guner et al. found that lateral TAP block performed at the end of laparoscopic donor nephrectomy significantly decreased pain score and total opioid consumption in the first 24 h [11, 12]. Similarly, Hosgood et al. also claimed that TAP block reduced the early morphine requirement (within 6 h after surgery) in a similar patient population [10]. It should be noted that there were two important differences between our study and the above-mentioned others. First, the location of the main surgical incision for kidney retrieval and trocar sites in the present study was completely different from those in the previous studies [10, 11]. Second, we performed TAP block before surgery, whereas others performed TAP block after surgery [11, 12]. Thus, when comparing results among different trials, it is crucial to take the surgical technique, the block approach and the time of block into consideration. Full clarification of our negative findings requires detailed understanding of the innervation of the abdominal wall.

The anterolateral abdominal wall is mainly innervated by the anterior rami of thoracolumbar spinal nerves (T6-L1), which follow a curvilinear course from the back towards the midline of the body [6]. Generally, as they proceed, after giving off lateral cutaneous branches near the costal angle innervating the lateral areas of the abdominal wall [14], they enter into the TAP with a varied course and finally perforate the rectus abdominis and end as the anterior cutaneous branches innervating the anterior abdomen (area from the midline to the midclavicular line) [15]. Most of the lateral cutaneous branches arise before the main nerves enter the TAP, and only those of T11 and T12 have a short course within or through the TAP [15]. Thus, it is not surprising that lateral TAP block can reliably provide analgesia for the lower anterior abdomen but not the lateral abdomen wall. Ma et al. further confirmed this by detecting the blocking dermatomes in 19 areas of the abdominal wall after lateral TAP block [16]. This might be an important reason for our negative results. The dermatomes of lateral TAP block we performed only covered part of the wounds in RLRS. For urological surgery, the posterior TAP approach [13, 17] or quadratus lumborum block [18] may be the better choice because they can block the lateral cutaneous branches of thoracolumbar spinal nerves and provide better lateral abdominal wall analgesia.

Regarding postoperative recovery, we observed no advantages in Group T in terms of the incidence of PONV. This was understandable since the perioperative opioid consumptions were comparable in the two groups. Similar findings were also reported in other studies [10, 19]. We found rather low sleep quality in both groups with no significant difference. We believed that not only pain intensity but also the surrounding environment in the ward affected sleep quality. Given that none of the above variables were different, it was no wonder that the length of hospital stay was similar in the two groups.

Our study has some limitations. First, we did not assess sensory dermatome blockage to confirm a successful TAP block because the block was performed after anaesthesia induction for blinding. Second, we only collected opioid consumption at a single time point (24 h after surgery) during the follow-up periods. Since we didn’t record the opioid consumption at earlier postsurgical time-points, such as at 6 and 12 h after surgery, we could not analyse the early effect of TAP block. Third, the range of 95% confidence interval of the median difference in opioid consumption between the two groups were large, which indicated that the present trial might be underpowered.

## Conclusions

Results of this prospective, randomized, double-blind trial showed that preoperative single-shot lateral TAP block did not decrease intra- and postoperative opioid consumption, nor did it relieve pain intensity or promote postoperative recovery in the first 24 h after surgery for patients undergoing retroperitoneoscopic renal surgery. However, considering the wide range of confidence interval of median difference in opioid consumption between the two groups, the trial might be underpowered.

## Supplementary information

**Additional file 1.** Original data. Dataset supporting the conclusions of this article.

## Data Availability

The dataset supporting the conclusions of this article is included within the article and its additional files.

## Abbreviations

ASA: America Society of Anaesthesiologists
BIS: Bispectral Index
BMI: Body mass index
COPD: Chronic obstructive pulmonary disease
NRS: Numeric rating scale
NSAIDs: Non-steroidal anti-inflammatory drugs
NYHA: New York Heart Association
PACU: Post-anaesthesia care unit
PCA: Patient-controlled analgesia
PONV: Postoperative nausea and vomiting
RLRS: Retroperitoneal laparoscopic renal surgery
TAP: Transversus abdominis plane
